# Intraoperative performance and influencing factors in computer-assisted total shoulder arthroplasty: a large-scale analysis

**DOI:** 10.1038/s41598-025-24124-2

**Published:** 2025-11-19

**Authors:** François Boux de Casson, Gabriella Ode, Ari R. Youderian, Bruno B. Gobbato, Alexandre Caubère, Amaury Jung, Sandrine Polakovic, Josie Elwell

**Affiliations:** 1 Blue Ortho, R&D, Meylan, France; 2 HSS Sports Medicine Institute, Hospital for Special Surgery, New York, NY USA; 3 South County Orthopedic Specialists, Laguna Woods, CA USA; 4 Department of Anatomy, Idomed University Jaragua, Jaragua do Sul, Brazil; 5 Hôpital National d’Instruction des Armées Sainte-Anne, Toulon, France; 6 Exactech, Gainesville, Florida USA

**Keywords:** Computational models, Biomedical engineering, Health care

## Abstract

The adoption of guidance technologies in total joint arthroplasty has expanded greatly in recent decades. This study aims to retrospectively evaluate the intra-operative performance of computer-assisted navigation (CAN) total shoulder arthroplasty (TSA) over years, analyzing temporal performance evolution and influencing factors. Over 40,000 cases utilizing a single CAN system (Exactech GPS) across an 8-year span were analyzed. We investigated the registration and navigation phases for anatomic (aTSA) and reverse (rTSA) procedures, focusing on factors influencing performance and temporal evolution. Data were stratified by surgeon volume, native anatomy, implant type, and procedural workflow. Key findings include: (1) Navigation times were longer for rTSA (mean 15:42 min) compared to aTSA (mean 10:01 min). (2) Glenoid retroversion > 20° and inferior inclination increased both registration and navigation durations for aTSA and rTSA. (3) Augmented implants extended navigation time for aTSA but not rTSA. (4) Surgeon experience significantly influenced performance; high-volume surgeons demonstrated markedly faster times, with a mean navigation time reduction of up to 7:16 min (aTSA) and 13:52 min (rTSA) compared to low-volume counterparts. (5) Registration time decreased over the years for all surgeon profiles, indicating system learning curves and increasing user familiarity. Additionally, repeated registration prolonged both registration and navigation phases, suggesting an impact on operator confidence or tracker visibility issues. Navigation performance trends over the years showed initial increases for aTSA due to added navigation steps but subsequent reductions for high- and medium-volume surgeons, underscoring the evolving efficiency of system use. The study highlights key factors influencing CAN efficiency and supports its potential to improve surgical accuracy and reduce variability, even among low-volume surgeons. This large-scale analysis underscores the utility of CAN in optimizing TSA workflows while identifying areas for potential improvement, particularly for complex anatomical cases.

## Introduction

Computer-assisted navigation (CAN) has been used for decades for total knee^1,2^ and hip^3^ arthroplasties and has only been available for total shoulder arthroplasty (TSA) for less than 10 years, but showing very rapid adoption^4^. Navigation for TSA currently allows only the glenoid side to be guided intra-operatively. Prior to CAN, computed tomography-based preoperative planning facilitated detailed assessment of shoulder morphology through 3D visualization. This approach enables precise evaluation of parameters such as glenoid version and inclination, optimizing the selection and positioning of implant components relative to the bony anatomy. CAN takes 3D visualization further by using real-time intraoperative visual guidance to execute the predetermined preoperative plan after a registration step. The navigation workflow differs between anatomic (aTSA) and reverse (rTSA) procedures. These guidance systems offer several proven benefits such as improving the accuracy of glenoid component placement, and precise correction of glenoid deformity, which is critical in preventing early implant loosening and failure^5–8^. Large scale studies assessing CAN in TSA has demonstrated low complication rates^9^. CAN has also demonstrated improvement in several outcome measures compared to matched cohort of non-navigated patients at 2 years follow-up, without any increased risk of complications^10,11^.

While several studies have evaluated the impact of the CAN on the overall duration in TSA, to our knowledge no study has assessed the temporal performance of the use of the CAN itself, i.e. the time required intraoperatively to perform the registration and navigation steps. The studies that have assessed the impact of this practice on the global operative time showed inconsistent results: some have reported that CAN lengthens the duration of the operation^12–14^ while others have reported the opposite^15–17^. However, the performance of surgeons when using these systems has not been studied in detail, nor have the factors that may influence this performance.

The purpose of this study was to retrospectively assess the time spent to perform registration and navigation phases using a single CAN system. We assessed the evolution of temporal performance of the registration and navigation, over an 8-year period since the launch of the system and investigated the factors influencing it. The performance of anatomical and reversed TSA CAN was evaluated separately, stratified according to operator profile and several criteria such as native anatomic measurements, implant type or workflow.

## Methods

### Patient cohort

Prospectively collected records from a single navigation system (Exactech GPS, Blue-Ortho, Meylan, France) using the same platform prosthesis (Equinoxe; Exactech, Gainesville, FL, USA) were retrospectively identified. Initially, all TSA cases performed worldwide using this CAN system since its inception in 2016 through 2024 were included. The following exclusion criteria were then applied:

- Reports involving cadaveric studies;
- Reports in which different cases are loaded one after the other (misuse);
- Reports with registration process restarted after navigation steps (misuse);
- Reports with registration process longer than 20 min (outliers);
- Reports with navigation phase longer than 70 min (outliers).

This database only hosts de-identified data. It does not contain any protected health information. Therefore, this study was exempt from institutional review board approval.

### Data collection and analysis

In the database, surgeons are distinguished using unique de-identified tokens. For each case, an operator profile was calculated based on the number of cases performed per sliding year (from date to date), and were divided into three surgeon profiles:

- Less than 10 cases: Low volume
- From 10 to 50: Medium volume
- More than 50: High volume

That is to say that a single operator could be classified as low, medium or high volume, depending on the dynamic of her/his activity over time.

Two performances were computed, in minutes, for all cases:

- Registration time: from the first acquisition to the exit of the registration check step. In between, a set of acquisitions recorded areas around the glenoid and the coracoid process. The registration steps are the same for aTSA and rTSA;
- Navigation time: from the entry point navigation until the exit of the last navigated step. The navigated steps are: (1) the entry point, (2) reaming, (3) center hole drilling, (3) peripheral hole drilling (only aTSA) or (3) screws lengths and directions (from 1 to 6 screws) (only rTSA) and (4) implant insertion^18^.

These performance times were also then stratified based on procedure type (aTSA or rTSA). And, separately for aTSA and rTSA procedures, the impact of the following factors was investigated:

- The native retroversion (less or greater to 20º);
- The native inclination (inf. or sup.);
- The implant type (augmented or not);
- The surgeon profile;
- Repeating the registration phase (one or several times).

Finally, changes in performance over the study duration for registration and navigation phases were assessed for all cases and according to the operator volume profiles.

### Statistical analysis

Data normality was tested using Shapiro-Wilk test. Equality of variances was tested using the Levene’s test. If the data exhibited normality with equal variances, the standard Student’s *t* test was applied. For normally distributed data with unequal variances, Welch’s *t* test was utilized. In cases where the data did not meet normality assumptions, the non-parametric Mann–Whitney U test was employed. Significance was set at the 5% level for all tests. Statistical analysis were performed using the Python SciPi v1.11.4 library^19^.

## Results

In total, 40,117 cases, performed by 1248 surgeons were included, comprising 7750 aTSA and 32,367 rTSA. Mean registration time was 3:26 ± 2:43 min and mean navigation time was 14:36 ± 9:24 min.

For the registration phase, there was no difference in performance between aTSA and rTSA (*p* = 0.699). For navigation phase, aTSA procedures were faster (10:01 ± 7:24 vs. 15:42 ± 9:30, *p* < 0.001). See Table 1. 

**Table 1:** Influence of procedure type on performance.

	aTSA		rTSA		*p* value
	N = 7750		N = 32367		Mann–Whitney
	Mean±SD	Median	Mean±SD	Median	
Registration (min)	3:23 ± 2:33	2:31	3:27 ± 2:45	2:32	*p* = 0.699
Navigation (min)	10:01 ± 7:24	7:48	15:42 ± 9:30	13:28	*p* < 0.001

For both aTSA and rTSA, native retroversion of the glenoid greater than 20° slowed the registration time (for aTSA: 3:43 ± 2:45 vs. 3:19 ± 2:30, *p* < 0.001, for rTSA: 3:35 ± 2:45 vs. 3:25 ± 2:45, *p* < 0.001) and the navigation time (for aTSA: 12:08 ± 8:17 vs. 9:37 ± 7:09, *p* < 0.001, for rTSA: 19:26±11:30 vs. 15:07 ± 8:59, *p* < 0.001). See Table 2.

**Table 2:** Influence of the glenoid native version on performance.

aTSA	Retroversion < 20°		Retroversion > 20°		*p* value
	Mean±SD	Median	Mean±SD	Median	Mann–Whitney
	N = 6444		N = 1264		
Registration (min)	3:19 ± 2:30	2:28	3:43 ± 2:45	2:45	*p* < 0.001
Navigation (min)	9:37 ± 7:09	7:26	12:08 ± 8:17	9:49	*p* < 0.001

rTSA	Retroversion < 20°		Retroversion > 20°		*p* value
	Mean±SD	Median	Mean±SD	Median	Mann–Whitney
	N = 27983		N = 4274		
Registration (min)	3:25 ± 2:45	2:31	3:35 ± 2:45	2:39	*p* < 0.001
Navigation (min)	15:07 ± 8:59	13:03	19:26 ± 11:30	16:28	*p* < 0.001

For both aTSA and rTSA, native glenoid inferior inclination was associated with prolonged registration time (for aTSA: 3:26 ± 2:34 vs. 3:18 ± 2:31, *p* = 0.002, for rTSA: 3:31 ± 2:48 vs. 3:24 ± 2:43, *p* < 0.001) and navigation time (for aTSA: 10:30 ± 7:46 vs. 9:26 ± 6:52, *p* < 0.001, for rTSA: 16:17 ± 10:04 vs. 15:21 ± 9:07, *p* < 0.001). See Table 3.

**Table 3:** Influence of the glenoid native inclination on performance.

aTSA	Inclination < 0°		Inclination > 0°		*p* value
	Mean±SD	Median	Mean±SD	Median	Mann–Whitney
	N = 4301		N = 3407		
Registration (min)	3:26 ± 2:34	2:34	3:18 ± 2:31	2:28	*p* = 0.002
Navigation (min)	10:30 ± 7:46	8:09	9:26 ± 6:52	7:24	*p* < 0.001

rTSA	Inclination < 0°		Inclination > 0°		*p* value
	Mean±SD	Median	Mean±SD	Median	Mann–Whitney
	N = 11369		N = 20885		
Registration (min)	3:31 ± 2:48	2:35	3:24 ± 2:43	2:31	*p* < 0.001
Navigation (min)	16:17 ± 10:04	13:51	15:21 ± 9:07	13:15	*p* < 0.001

For aTSA, use of the augmented implant slowed both registration (3:29 ± 2:39 vs. 3:11 ± 2:20, *p* < 0.001) and navigation times (10:44 ± 7:50 vs. 8:42 ± 6:18, *p*< 0.001). For rTSA, on the contrary, use of the augmented implant slightly accelerated registration (3:23 ± 2:44 vs. 3:34 ± 2:48, *p* < 0.001) but had no impact on navigation (*p* = 0.34). See Table 4.

**Table 4:** Influence of the implant type (augmented or not) on performance.

aTSA	Augmented		Non augmented		*p* value
	Mean±SD	Median	Mean±SD	Median	Mann–Whitney
	N=5022		N=2728		
Registration (min)	3:29 ± 2:39	2:35	3:11 ± 2:20	2:24	*p* < 0.001
Navigation (min)	10:44 ± 7:50	8:25	8:42 ± 6:18	6:51	*p* < 0.001

rTSA	Augmented		Non augmented		*p* value
	Mean±SD	Median	Mean±SD	Median	Mann–Whitney
	N = 22335		N = 10032		
Registration (min)	3:23 ± 2:44	2:30	3:34 ± 2:48	2:38	*p* < 0.001
Navigation (min)	15:35 ± 9:15	13:27	15:57 ± 10:00	13:29	*p* = 0.34

**Fig. 1:**
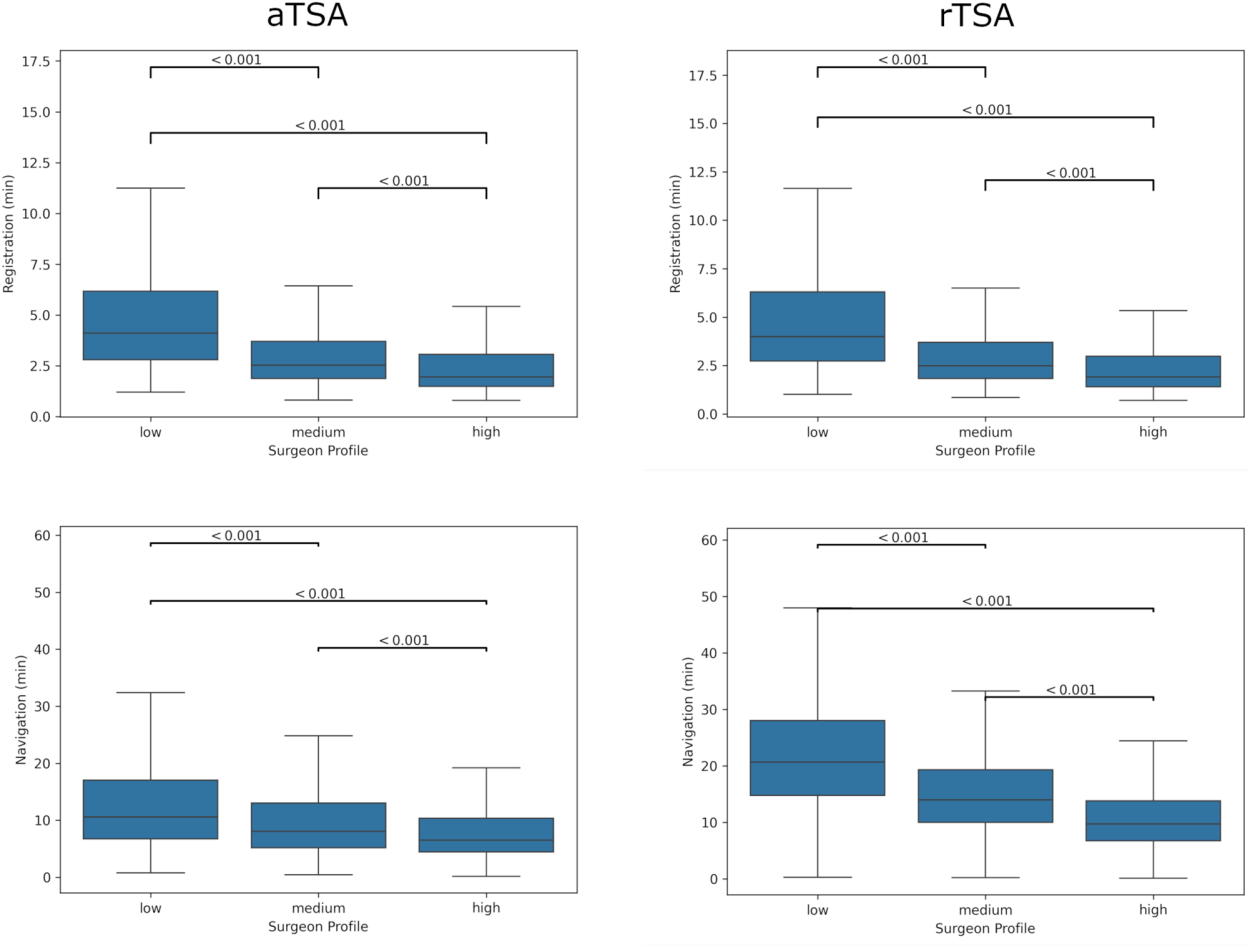
Intra-operative performance by surgeon profile.

Stratifying cases by surgeon volume, 9002 cases were performed by low volume surgeons, 16,687 by medium volume and 14,428 by high volume. Regarding the impact of surgeon profile on the performance, for aTSA, during the registration phase, high volume surgeons are 30 s faster than medium volume and 2:16 min faster than low volume, while medium volume surgeons are 1:45 min faster than low volume. For navigation, high volume surgeons are 1:57 min faster than medium volume and 5:00 min faster than low volume. Medium volume surgeons are 3:02 min faster than low volume. All differences were significant (*p* < 0.001). See Fig. 1.

For rTSA, during the registration phase, high volume surgeons were 39 s faster than medium volume and 2:32 min faster than low volume, while medium volume were 1:52 min faster than low volume. For navigation, high volume surgeon were 4:27 min faster than medium volume and 11:20 min faster than low volume. Medium volume surgeons were 6:53 min faster than low volume. All the differences were significant (*p* < 0.001).

For both aTSA and rTSA, repeating the registration phase several times significantly slowed the registration time (for aTSA: 5:55 ± 3:13 vs. 2:51 ± 2:01, *p* < 0.001, for rTSA: 6:28 ± 3:44 vs. 2:45 ± 1:54, *p* < 0.001) but also the navigation time (for aTSA: 11:19 ± 8:19 vs. 9:45 ± 7:10, *p* < 0.001, for rTSA: 19:15 ± 10:48 vs. 14:54 ± 8:59, *p* < 0.001). See Table 5.

**Table 5:** Influence of repeated registration on performance.

aTSA	One		Several		*p* value
	Mean±SD	Median	Mean±SD	Median	Mann–Whitney
	N = 6415		N = 1335		
Registration (min)	2:51 ± 2:01	2:14	5:55 ± 3:13	5:05	*p* < 0.001
Navigation (min)	9:45 ± 7:10	7:34	11:19 ± 8:19	8:56	*p* < 0.001

rTSA	One		Several		*p* value
	Mean±SD	Median	Mean±SD	Median	Mann–Whitney
	N = 26374		N = 5993		
Registration (min)	2:45 ± 1:54	2:14	6:28 ± 3:44	5:23	*p* < 0.001
Navigation (min)	14:54 ± 8:59	12:49	19:15 ± 10:48	17:06	*p* < 0.001

**Fig. 2:**
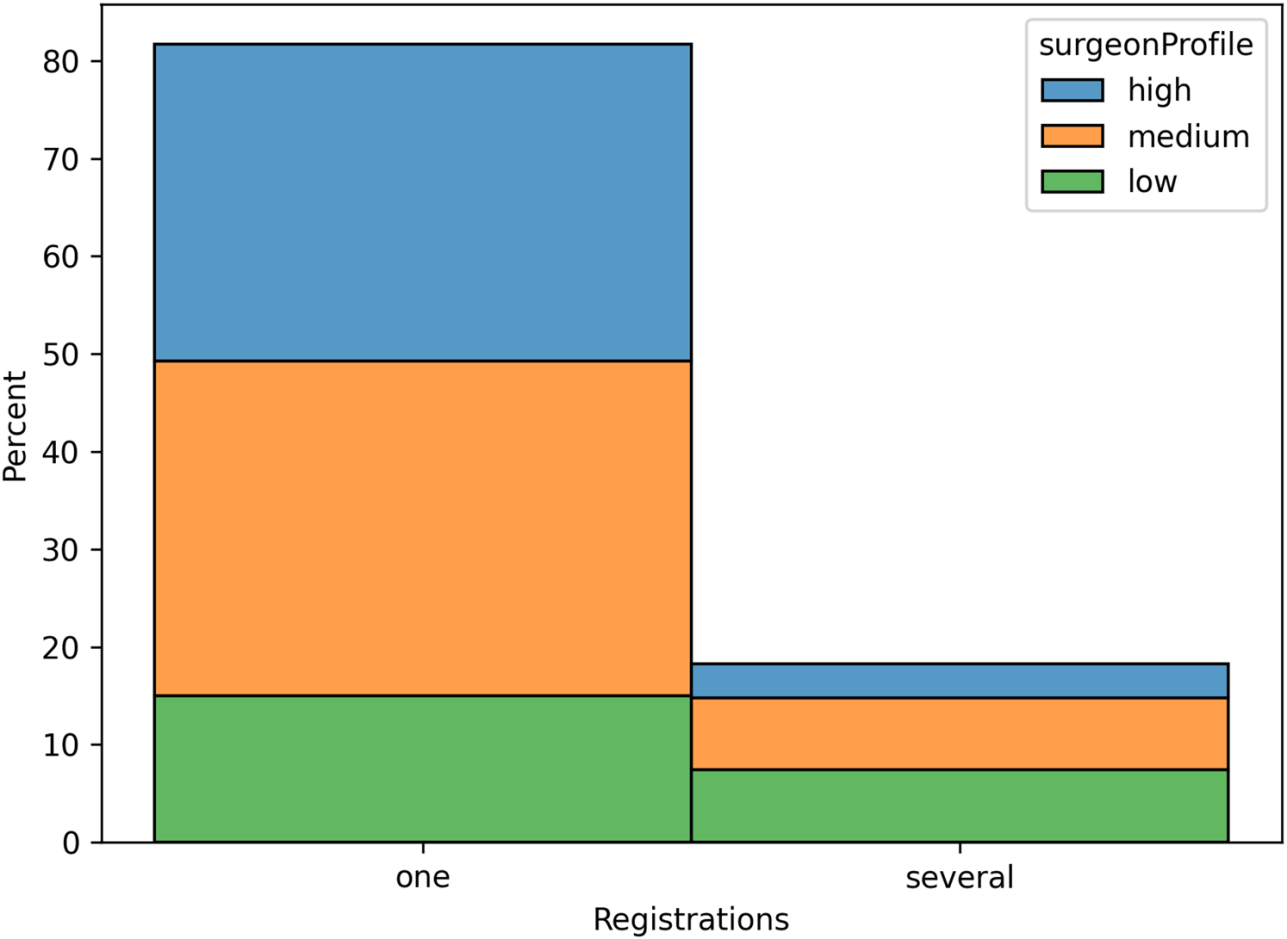
Influence of the surgeon’s profile on the number of registrations.

For both aTSA and rTSA, the majority of registrations (81.7%) were completed on the first try. Surgeon volume correlated with a higher likelihood of completing registration on the first try: 90.3% for high volume surgeons, 82.4% for medium volume and 66.8% for low volume, see Fig. 2.

**Fig. 3:**
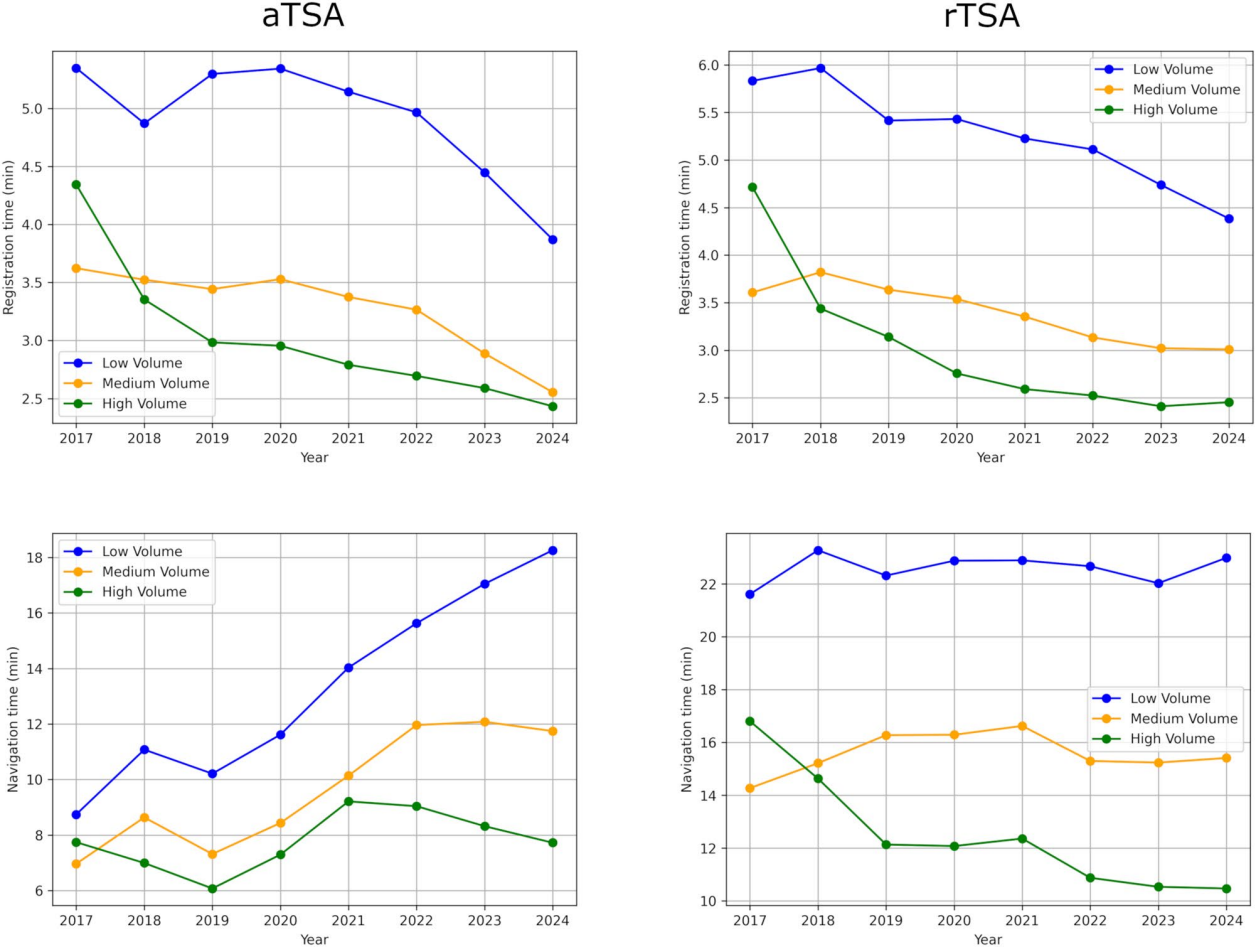
Evolution of the intra-operative performance, over the years.

Over the years, from 2017 to 2024, all surgeons included, registration time decreased continuously from 4:30 to 2:36 min for aTSA and from 4:46 to 2:59 min for rTSA. Navigation time lengthened from 7:52 to 10:11 min for aTSA but shortened from 18:04 to 14:31 min for rTSA. These trends were shared by all surgeon volume profiles. See Fig. 3.

## Discussion

This large-scale study on a single CAN system investigated the factors influencing the temporal performance of the intra-operative workflow associated with using the CAN system and the evolution of this performance. Most notably, for both aTSA and rTSA, glenoids with high native retroversion or inferior inclination had longer navigation phases. Repeating the registration phase also extends navigation time. Navigating augmented implants took longer for aTSA but not for rTSA. Finally, although a higher volume of cases performed by year increases operators’ performance, all surgeons gained in efficiency over the years, even low-volume profiles.

Comparison of our findings with existing studies is challenging due to the limited availability of shoulder navigation guidance systems on the market. To our knowledge, no studies specifically addressing the temporal performance of these systems have been published. For total knee arthroplasty, using a navigation system showed an additional time of + 14 min, similar to an image-based robotic system but faster than imageless robotic assistance (+ 25 min)^20^. For TSA, as previously mentioned, results are more mixed, and the factors influencing this performance have not previously been evaluated. Hones et al. demonstrated that navigation for rTSA reduced the number of screws, thus decreasing operative time, but did not report quantitative time data^21,22^. Using another CAN system with head-up display, Dey Hazr et al. showed on a cadaveric study that rTSA surgical time increased from 10 (junior) to 18 min (senior)^23^. On the same system, Rojas et al. reported a prospective series with 17 rTSA cases showing mean registration time of 08:16 ± 04:54 with 3 cases requiring a second registration (17.6%, close to our findings) and a mean navigation time of 23:57^24^. These registration and navigation performance for rTSA are twice as long as in our study, which include 32,367 cases.

The different navigation performance between aTSA and rTSA is likely related to the fact that more steps are required for navigation for rTSA procedures. For aTSA procedures, the entry point and orientation of the k-wire, reaming and implant insertion (since 2019) are navigated. For reversed procedures, in addition to the aforementioned steps, screw insertions (from 1 to 6 screws) are also navigated.

Concerning influencing factors, we demonstrated that highly retroverted glenoids lead to longer procedures (registration and navigation) for both aTSA and rTSA procedures. This is likely due to the difficulty inherent to this type of morphology. Reduced visibility combined with challenging glenoid exposure may also explain the lower performance of cases with glenoids showing inferior inclination.

This study showed that using augmented implants extends the operating time for aTSA. This could be explained by the fact that these cases are likely performed in glenoids with greater pre-operative deformity which may lead to a more technically more challenging reaming step. On the other hand, using augmented implants had no performance impact on rTSAs, but navigation of the reaming step is only one step in seven for rTSAs, whereas it represents a third of the steps for aTSAs. The impact of a potentially longer reaming step for rTSA is therefore reduced.

Analysis of performance according to the number of cases operated on in the previous year showed that experience improves the temporal performance for both registration and navigation significantly, even for surgeons with medium volumes compared to those with low. The decrease in surgery time can reach 7:16 min between high and low profiles for aTSA and 13:52 in rTSA.

Repeated registrations obviously extend the registration time. But our study showed that this is also impacting the navigation duration. We evaluated if high retroversion or inferior inclination could increase the occurrence of repeated registration, but found no significant difference. As in all cases, 18% with these deformities were registered several times. The impact on the navigation time is probably linked either to a visibility issue (incorrect trackers installation) or to a loss of operator confidence. In any case, the user experience reduced the occurrence of repeated registrations (more than 33% of cases for low-volume profiles, less than 18% for medium-volume and less than 10% for high-volume profiles).

For both aTSA and rTSA, performance over the years showed a downward trend in registration times for all profiles, even low volume surgeons. This shows that either users are gaining in experience by using the system, even with reduced activity, or that the culture of navigation is progressing and favoring performance, but likely a combination of these two factors. The system’s short learning curve should also encourage this phenomenon^15^.

Concerning navigation performance, results over the last 8 years for aTSA are more mixed. After a phase of increasing navigation time between 2019 and 2022, this time tends to decrease for high- and medium-volume surgeons. This is potentially explained by the introduction of an implant rotation navigation step between 2019 and 2022. However, navigation time is still increasing for low-volume practitioners. Perhaps this is linked to the increasing use of rTSA versus aTSA, in which low-volume surgeons in particular are less practiced and confident performing aTSA procedures^25,26^.

The results of this study must be interpreted with the following limitations in mind. First, this is limited to a single navigation system. Second, the input data is limited to navigation application logs, with no patient demographic data, no measurement of overall operating times (from incision to closure), and no tracker placement duration. Third, we did not compare to cases where navigation was not used and therefore, we are not able to comment on impact of using navigation on overall surgical time or how these various factors may impact that in non-navigated cases. In other words, it’s possible that these same factors impact overall surgical time in non-navigated cases, but we do not know to what extent (perhaps the disparity between placing an augmented versus implant component is even greater when navigation is not used). Finally, some of the significantly different variables may not translate to clinically impactful differences (i.e. there are significant differences in registration time for the retroversion/inclination analyses, but in many cases these differences are less than 10 s).

A relevant follow-up to this work would be to investigate a possible link between the performance measured in this study and clinical scores or patient reported outcome measures.

## Data Availability

The data that support the findings of this study are published under CC BY 4.0 licence on https://figshare.com/. (doi:10.6084/m9.figshare.30148690)
